# Netrin‐1 alleviates subarachnoid haemorrhage‐induced brain injury via the PPARγ/NF‐KB signalling pathway

**DOI:** 10.1111/jcmm.14105

**Published:** 2019-01-07

**Authors:** Junhui Chen, Yong Xuan, Yan Chen, Ting Wu, Lei Chen, Haoxiang Guan, Shuo Yang, Jianqing He, Dongliang Shi, Yuhai Wang

**Affiliations:** ^1^ Department of Neurosurgery Wuxi Medical College of Anhui Medical University (l0lst Hospital of PLA) Wuxi City Jiangsu Province PR. China; ^2^ Department of Orthopedic The Second People's Hospital of Hefei Hefei City Anhui Province PR. China; ^3^ Department of Physical Examination Center Hexian Peoples Hospital Ma Anshan City Anhui Province PR. China; ^4^ Department of Cardiology Wuxi Medical College of Anhui Medical University (l0lst Hospital of PLA) Wuxi City Jiangsu Province PR. China

**Keywords:** brain injury, netrin‐1, PPARγ/NF‐KB signalling pathway, subarachnoid haemorrhage

## Abstract

Netrin‐1 (NTN‐1) is a novel drug to alleviate early brain injury following subarachnoid haemorrhage (SAH). However the molecular mechanism of NTN‐1‐mediated protection against early brain injury following SAH remains largely elusive. This study aims to evaluate the effects and mechanisms of NTN‐1 in protecting SAH‐induced early brain injury. The endovascular perforation SAH model was constructed using male C57BL/6J mice, and recombinant NTN‐1 was administrated intravenously. Mortality rates, SAH grade, brain water content, neurological score and neuronal apoptosis were evaluated. The expression of PPARγ, Bcl‐2, Bax and nuclear factor‐kappa B (NF‐κB) were detected by Western blot. Small interfering RNA specific to NTN‐1 receptor, UNC5B, and a selective PPARγ antagonist, bisphenol A diglycidyl ether (BADGE), were applied in combination with NTN‐1. The results suggested that NTN‐1 improved the neurological deficits, reduced the brain water content and alleviated neuronal apoptosis. In addition, NTN‐1 enhanced PPARγ and Bcl‐2 expression and decreased the levels of Bax and NF‐κB. However, the neuroprotection of NTN‐1 was abolished by UNC5B and BADGE. In conclusion, our results demonstrated that NTN‐1 attenuates early brain injury following SAH via the UNC5B PPARγ/NF‐κB signalling pathway.

## 1 INTRODUCTION

Clinical management of brain injury following SAH remains a challenge. A therapeutic strategy that effectively promotes brain tissue repair and regeneration is highly desirable. Netrin‐1 (NTN‐1) was initially discovered as a critical molecule during embryo development by providing key guidance cues for commissural axon development.^1^ NTN‐1 attracts or repels axonal growth cones by binding to receptors, including UNC5 (uncoordinated‐5) and DCC (Deleted in Colorectal Cancer).^2^ Recently, the neuroprotection of NTN‐1 has drawn increasing interest.^3^, ^4^ It has been suggested that NTN‐1 promotes neovascularization in brain by inducing the proliferation, migration and tube formation of human cerebral endothelial cells and human aortic smooth muscle cells.^5^ NTN‐1 was also found to exert immune regulatory function, which consequently fosters its broad clinical implications in non‐neural system diseases.^6^, ^7^, ^8^ It is also reported that NTN‐1 attenuates infiltration of immune cells into brain parenchyma.^9^ This evidence implicates the use of NTN‐1 as a potential mediator in autoimmune central nervous system disease. Moreover, neuronal apoptosis is a crucial pathological process in early brain injury after subarachnoid haemorrhage (SAH). NTN‐1 has been employed as an anti‐apoptosis molecule to alleviate the SAH‐induced brain injury.^10^

Despite the evidences, the role of NTN‐1 in alleviating brain injury still remains controversial. It was shown that spinal axons are unable to regenerate after injury, partly as a result of axon growth inhibition orchestrated by NTN‐1 expression and its interaction with the receptors. Therefore, NTN‐1 is also considered as a myelin‐associated inhibitor of axonal regeneration in neuronal injury. It is imperative to clarify the role of NTN‐1 in neuroregenerative therapies.

Peroxisome proliferator‐activated receptor gamma (PPARγ) is a critical transcription factor that mediates apoptosis, oxidative stress and inflammation in a variety of diseases. Evidence indicates that PPARγ is a downstream effector molecule of NTN‐1, which is indispensable for the NTN‐1 regulation of inflammation during ischaemia‐reperfusion (IR) injury in heart and kidney.^11^ Moreover, nuclear factor‐kappa B (NF‐κB) pathway was found to be inhibited by NTN‐1 treatment, whereby PPARγ is critical for the transduction of NTN‐1 binding to NF‐κB inhibition.^12^

Herein, we aim to clarify the effects of NTN‐1 treatment in SAH‐induced early brain injury. We focused on the role of PPARγ and NF‐κB in this process. To clarify the role of NTN‐1 receptors and PPARγ in this process, the UNC5B inhibitor and PPARγ antagonist were used in combination with NTN‐1 treatment, followed by evaluating brain water content, neurological score and apoptosis.

## 2 MATERIALS AND METHODS

### 2.1 Materials

Exogenous rh‐NTN‐1 (R&D Systems, USA), Bisphenol A diglycidyl ether (BADGE) (Sigma‐Aldrich, MO, USA), UNC5B siRNA and Scr siRNA (Thermo Fisher Scientific, USA) were used in this study. Terminal deoxynucleotidyl transferase dUTP nick‐end labelling (TUNEL) kits were purchased from Roche (Mannheim, Germany). Rabbit monoclonal antibodies against Sirt1, Bcl‐2, Bax, β‐actin, and rabbit polyclonal antibody against NF‐κB were acquired from Cell Signaling Technology (Beverly, MA, USA).

### 2.2 Animals

All animal studies were performed in accordance to protocols approved by the Ethics Committee of the Wuxi Medical College of Anhui Medical university (l0lst Hospital of PLA). All the experiments were conducted on healthy adult male C57BL/6J mice (22‐25 g) (Jackson Laboratories, USA). The mice were maintained under a pathogen‐free condition at about 22°C on a 12 hour light–dark cycle with free access to food and water.

### 2.3 Animal models

The endovascular perforation method was used to construct the SAH model based on a protocol that previously described.^13^ Briefly, animals were anaesthetized by intraperitoneal (i.p.) injection of 50 mg/kg pentobarbital sodium. Rectal temperature was kept at 37 ± 0.5°C during operation using a heating pad. A midline incision was made in the neck, and the left common, external and internal carotid arteries were exposed. Following this, the left external carotid artery was ligated and cut, leaving a 3‐mm stump. A 4–0 monofilament nylon suture was inserted into the left internal carotid artery through the external carotid artery stump to perforate the artery at the bifurcation of the anterior and middle cerebral artery. Mice in the sham group underwent the same procedures without the artery perforation.

Mice were randomly assigned to the following groups: (i) sham group, (ii) SAH group, (iii) SAH + NTN‐1 group, (iv) SAH + NTN‐1+ UNC5B siRNA group, (v) SAH + NTN‐1+ BADGE group, and (vi) SAH + NTN‐1+ scramble siRNA group. A quantity of 45 μg/kg NTN‐1 was intravenously injected into the mice at 2 and 12 hours after SAH. The SAH group and the sham group received equal volumes of PBS at the corresponding time points. BADGE was intraperitoneally injected at a dose of 30 mg/kg for a total of 6 days prior to SAH in the SAH + NTN‐1+ BADGE group.

### 2.4 siRNA treatment

Mice were anaesthetized with pentobarbital sodium (50 mg/kg) and placed on a stereotaxic apparatus (Narishige, Tokyo, Japan). Then the bregma point was exposed. A burr hole was made in the left hemisphere using the following coordinates: 0.2 mm posterior, 1 mm lateral, and 2.2 mm below the horizontal plane of the bregma. Two microliters of 1 μg/μL UNC5B‐specific or scramble siRNA were injected into the lateral ventricle with a specialized syringe (Hamilton Company, Reno, NV, USA). To enhance the silencing effect, the injection was performed at 12 and 24 hours before SAH.

### 2.5 Mortality and SAH grade

Mortality was documented at 24 hours after SAH. SAH grade was given according to a previously described grading system.^14^ Briefly, the grading was given based on subarachnoid blood blot: grade 0, no subarachnoid blood; grade 1, minimal subarachnoid blood; grade 2, moderate blood clot with appreciable arteries; and grade 3, blood clot obliterating all arteries within the segment. The grade ranges from 0 to 18. Mice with SAH grading scores of less than 7, which had no prominent brain injury, were excluded from the study.

### 2.6 Neurological score

The severity of brain injury was also evaluated based on neurological score given at 24 hours after SAH.^15^ Neurological deficits were yielded based on an 18‐point system consisting of six tests on spontaneous activities (0‐3), movement symmetry of all limbs (0‐3), body proprioception (1‐3), forelimbs outstretching (0‐3), climbing (1‐3), and response to vibrissae touch (1‐3). The neurological score ranges from 3 to 18. A higher score is representative of a better neurological function.

### 2.7 Brain water content

Brain water content was quantified by the standard wet–dry method.^16^ At 24 hours after SAH, mice were killed, and brains were immediately harvested. Brain specimens were separated into the left and right cerebral hemispheres, followed by weighting cerebellum and brain stem (wet weight). Following this, brain specimens were dehydrated at 105°C for 24 hours to acquire the dry weight. Brain water content was calculated as follows: (wet weight‐dry weight)/wet weight × 100%.

### 2.8 TUNEL staining

TUNEL assay was conducted to assess apoptosis in the brain cortex. TUNEL reaction mixture (50 μL) was added on each sample, and the slides were incubated in a humidified dark chamber for 60 minutes at 37°C. The slides were then incubated with DAPI for 5 minutes at room temperature in the dark to stain the nuclei, followed by imaging with a fluorescence microscope. Apoptotic index was expressed as the ratio of the number of TUNEL‐positive neurons to the total number of neurons in the field of view.

### 2.9 Western blot

The left basal cortical sample was collected and homogenized for protein extraction. Protein lysates were resolved by sodium dodecyl sulphate‐polyacrylamide gel electrophoresis, followed by transferring onto Immobilon nitrocellulose membranes (Millipore, Boston, MA, USA). The membranes were blocked with 5% non‐fat milk in TBS‐T (Tween 20, 0.1%) at room temperature. Rabbit antibodies against PPARγ, NF‐κB, Bcl‐2, Bax and β‐actin (1:1000) were applied to the membrane and incubated overnight at 4°C. After washing with TBST, horseradish peroxidase‐conjugated anti‐rabbit IgG secondary antibodies (1:5000) were applied to the membrane and incubated at room temperature for 1.5 hours. The protein bands were acquired, using a Bio‐Rad imaging system (Bio‐Rad, Hercules, CA, USA) and quantified with ImageJ.

### 2.10 Statistical analysis

SPSS 18.0 and GraphPad Prism 6 were used for statistical analysis. Mortality analysis was performed, using the Fisher's exact test. SAH grades and neurological scores were expressed as median and 25th to 75th percentiles, which were analysed by Kruskal‐Wallis one‐way analysis of variance (ANOVA) on ranks or the Mann‐Whitney *U* test, followed by Dunn's or Tukey post hoc analysis. Other data are presented as the mean ± SD. Multiple group comparisons were tested by one‐way ANOVA. Intergroup comparisons were made by Tukey post hoc analysis. Differences with *P* < 0.05 were considered as statistically significant.

## 3 RESULTS

### 3.1 Treatment with NTN‐1, UNC5B siRNA or BADGE has no long‐term effects on mortality rates and SAH grade in SAH models

**Figure 1:**
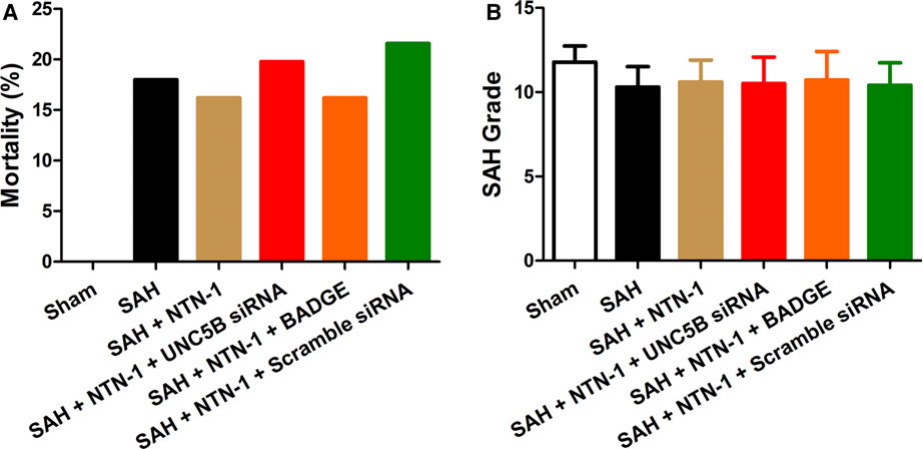
Treatment with NTN‐1, UNC5B siRNA or BADGE has no effects on mortality rates and SAH grade in SAH models. A, Mortality rates in the SAH group (8%), the SAH + NTN‐1 group rate (16.2%), the SAH + NTN‐1+ UNC5B siRNA group rate (19.8%), the SAH + NTN‐1+ BADGE group (16.2%), and the SAH + NTN‐1+ scramble siRNA group (21.6%). B, The SAH grade scores in the SAH group, the SAH + NTN‐1 group, the SAH + NTN‐1+ UNC5B siRNA group, the SAH + NTN‐1+ BADGE group, and the SAH + NTN‐1+ scramble siRNA group, which showed no significant differences (one‐way ANOVA analysis)

Previous studies indicated that the neuroprotective effects of NTN‐1 were evident at early time points after SAH, but the long‐term neuroprotection of NTN‐1 was unclear.^12^ We first evaluated the effect of NTN‐1 treatment on long‐term neurological damage parameters, including mortality rates and SAH grades. As shown in Figure 1, mortality rates (Figure 1A) and SAH grades (Figure 1B) in various groups, including SAH, SAH+NTN‐1, SAH+NTN‐1+ UNC5B siRNA, SAH+NTN‐1+BADGE, or SAH+NTN‐1+ scramble siRNA did not significantly differ, suggesting that NTN‐1 treatment has no effects in alleviating SAH in the long‐term. Based on this, we focused on evaluating the therapeutic effects of NTN‐1 treatment on early brain injury in the following studies.

### 3.2 NTN‐1 treatment exerts neuroprotective effects in early brain injury

**Figure 2:**
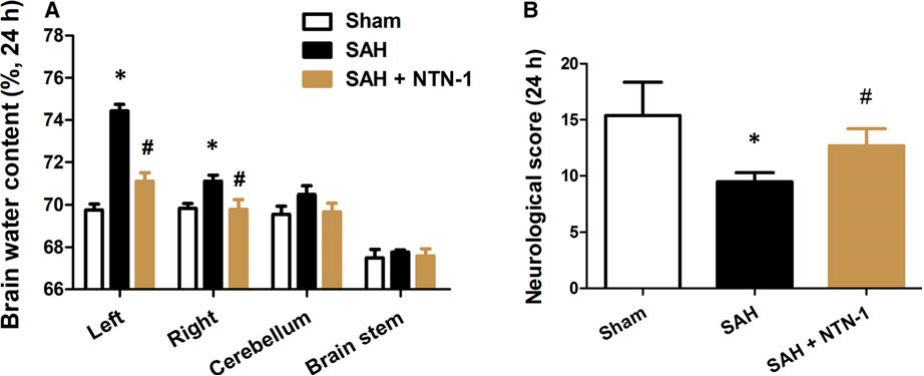
NTN‐1 treatment decreases brain water content and improves neurological score at 24 h after SAH. Evaluation of (A) brain water content and (B) neurological score of mice in Sham group, SAH group and SAH mice treated with NTN‐1 at 24 h after SAH (n = 6). *P < 0.05 vs sham group, #*P* < 0.05 vs SAH group

Brain water content and neurological scores, measured at 24 h after SAH, were used as early brain injury indicators of the neuroprotective effects of NTN‐1 treatment. As shown in Figure 2A, while SAH markedly increased brain water content in left and right hemisphere (*P* < 0.05), NTN‐1 treatment attenuated the elevation of brain water content (*P* < 0.05). Neurological scores, which were decreased by SAH, demonstrated significant improvement by NTN‐1 treatment (*P* < 0.05) (Figure 2B).

**Figure 3:**
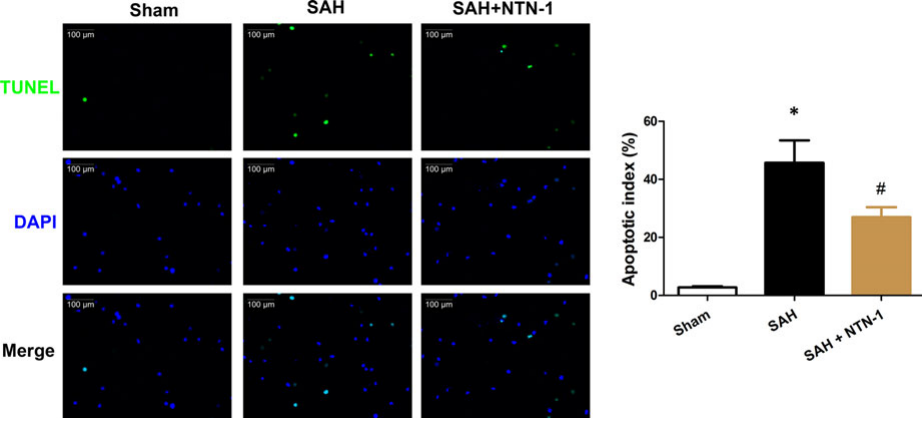
NTN‐1 alleviates neuronal apoptosis in the brain cortex 24 h after SAH. Representative images of apoptotic neurons are shown. Scale bar = 100 μm. The data are presented as the mean ± SEM (n = 6). *P < 0.05 vs sham group, #*P* < 0.05 vs SAH group

We hypothesized that the improved neurological scores were results of apoptosis reduction in brain cortex. Therefore, TUNEL assay was used to quantify the level of apoptosis in treated and untreated SAH mice at 24 hours after model construction. Indeed, our results showed that SAH led to increased apoptosis in brain cortex, which was prominently decreased by NTN‐1 treatment (Figure 3). We further showed that NTN‐1 treatment attenuates changes in the expressions of caspase‐3, cleaved caspase‐3, caspase‐9 and cleaved caspase‐9, which were cellular markers of apoptosis (Figure S1C,D). Collectively, these data corroborated the neuroprotective effects of NTN‐1 treatment.

### 3.3 The protective effect of NTN‐1 is mediated by UNC5B/ PPARγ/NF‐κB pathway

**Figure 4:**
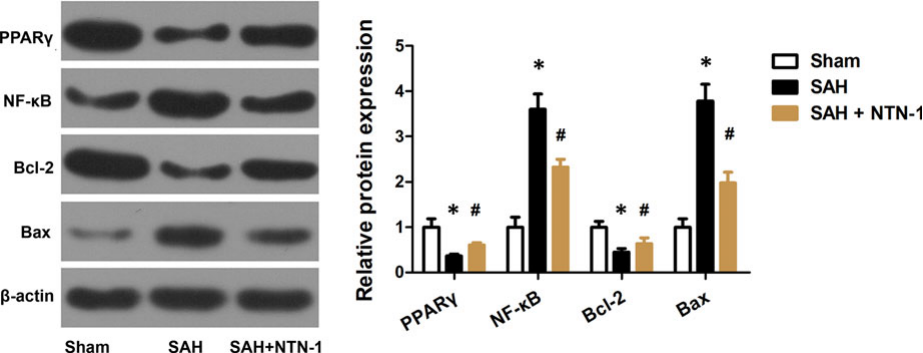
NTN‐1 treatment attenuates changes in the expressions of PPARγ, NF‐κB, Bcl‐2 and Bax. Representative Western blot gel image and quantitative analysis of the PPARγ, NF‐κB, Bcl‐2, and Bax expression were shown (n = 6). *P < 0.05 vs sham group, #*P* < 0.05 vs SAH group

To clarify the mechanism of the neuroprotective effects of NTN‐1 in early brain injury, we first explored whether NTN‐1 treatment alters the expression of PPARγ, NF‐κB, Bcl‐2 and Bax, which were markers of inflammation and apoptosis.^12^, ^17^ As shown in Figure 4, SAH induced reduction in PPARγ and Bcl‐2 and upregulation in NF‐κB and Bax.

**Figure 5:**
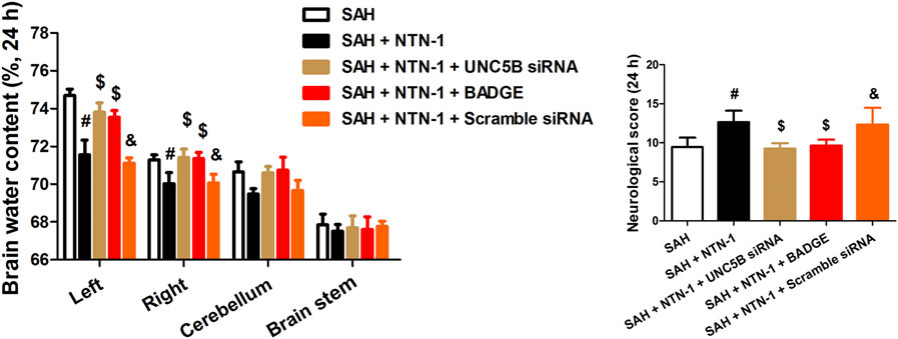
NTN‐1 treatment improves brain water content and neurological scores at 24 h after SAH, which is abrogated by UNC5B siRNA, and BADGE treatment. A, Brain water content of left and right hemispheres, cerebellum and brain stem harvested from different groups (24 h after SAH). B, Neurological scores of different groups (24 h after SAH). The data are presented as the mean ± SD, n = 6 for each group. #*P* < 0.05 vs SAH group, $*P* < 0.05 vs SAH + NTN‐1 group, &*P* < 0.05 vs SAH + NTN‐1+ UNC5B siRNA group

**Figure 6:**
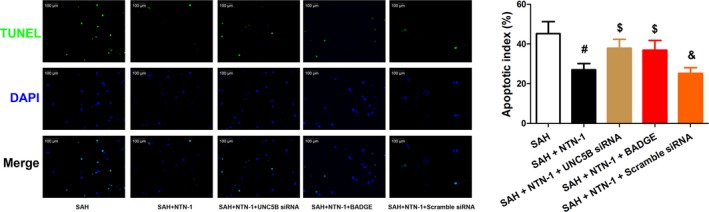
NTN‐1 treatment reduces neuronal apoptosis in the brain cortex at 24 h after SAH, which is abrogated by UNC5B siRNA, and BADGE treatment. Representative TUNEL staining fluorescent images of apoptotic neurons, and quantification of the apoptotic index are shown. Scale bar = 100 μm (n = 6). #*P* < 0.05 vs SAH group, $*P* < 0.05 vs SAH + NTN‐1 group, &*P* < 0.05 vs SAH + NTN‐1+ UNC5B siRNA group

Further, to validate this finding, the siRNA specific to NTN‐1 receptor, UNC5B, and a selective PPARγ antagonist, BADGE, were applied in combination with NTN‐1 treatment. We first showed that UNC5B siRNA efficiently knocked down UNC5B expression (Figure S1E,F). Unsurprisingly, the improvement on brain water content (in left and right hemispheres) and neurological scores by NTN‐1 treatment was greatly abrogated by UNC5B siRNA and BADGE (Figure 5A,B). Similarly, the alleviation of neuronal apoptosis in brain cortex by NTN‐1, at 24 hours after SAH, was counteracted by UNC5B siRNA and BADGE (Figure 6). As a control, the scramble siRNA did not cause apparent changes on the neuroprotective effects exerted by NTN‐1.

**Figure 7:**
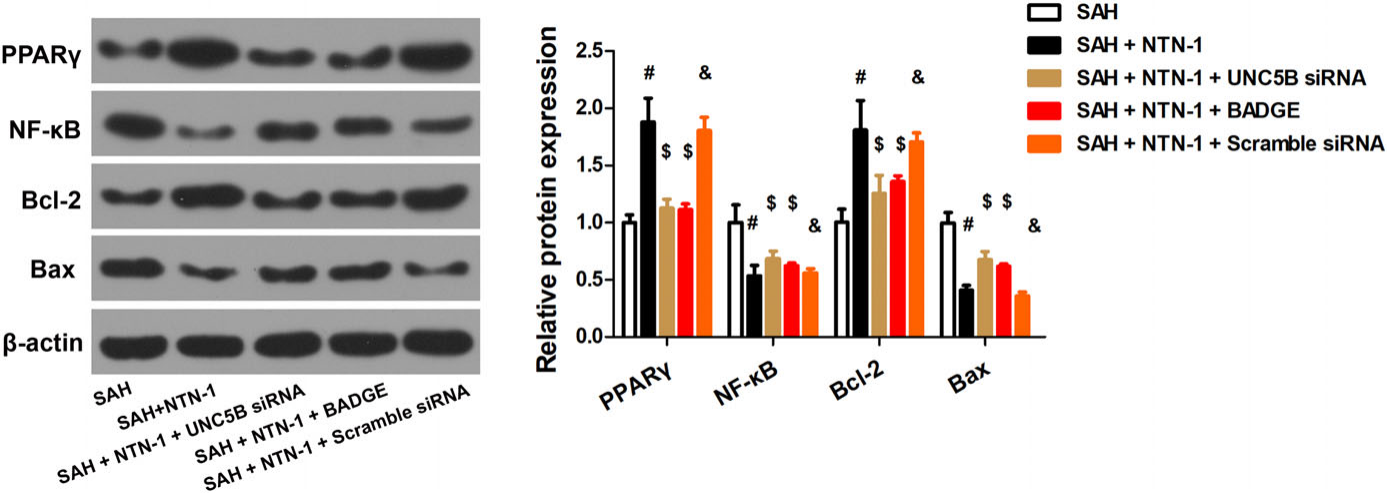
The protective effect of NTN‐1 is mediated by UNC5B/PPARγ/NF‐κB pathway. Representative Western blot gel image of PPARγ/NF‐κB and corresponding protein quantification are shown (n = 6). #*P* < 0.05 vs SAH group, $*P* < 0.05 vs SAH + NTN‐1 group, &*P* < 0.05 vs SAH + NTN‐1+ UNC5B siRNA group

Correspondingly, we assessed PPARγ, NF‐κB, Bcl‐2, and Bax expression in the presence of UNC5B siRNA and BADGE. As shown in Figure 7, UNC5B siRNA and BADGE attenuated the expression changes in PPARγ, NF‐κB, Bcl‐2, and Bax brought by NTN‐1 treatment. All these evidences confirmed that the protective effect of NTN‐1 is mediated by UNC5B/ PPARγ/NF‐κB pathway.

## 4 DISCUSSIONS

Here, we evaluated the therapeutic potential of a recombinant protein, NTN‐1, in alleviating early brain injury in a mouse SAH model. Following SAH, neural cell apoptosis plays a critical role in the development and aggravation of brain injury, concomitant with release of pro‐inflammatory mediators, leucocyte infiltration, resident immune cells activation and elevated apoptosis.^18^ As a result, patients suffer from disruption of blood‐brain barrier (BBB), brain oedema, worsening of cognitive functions, etc.^13^ Our study is preceded by a number of reports on treatment with exogenous NTN‐1, which can efficiently alleviate inflammation in non‐neural diseases.^7^, ^19^, ^20^ Our data consolidated that the effects of NTN‐1 treatment in SAH‐induced early brain injury are beneficial, evidenced by reduced brain oedema and improved neurological score. Apoptosis of neural cells was greatly alleviated. It is worth noting that long‐term outcomes of the treatment, including mortality and SAH grade, have not shown improvement. However, in other brain injury models, such as transient focal ischaemia,^5^ long‐term effects of NTN‐1 has been observed. As such, further studies are warranted to explain these results, and a combinatorial therapy strategy needs to be adopted to enhance the therapeutic outcome in SAH.

We further showed that the interaction of NTN‐1 with its receptor, UNC5B, is indispensable to alleviate in SAH‐induced early brain injury, which can be abolished by the inhibitors of UNC5B receptors. These findings are supported by previous studies showing that the binding of NTN‐1 to UNC5B receptor is paramount to attenuating inflammation and suppressing leucocyte infiltration.^21^ In line with this, neutralization of the UNC5B receptor with blocking antibodies was able to abolish the ameiliorating effects of NTN‐1 on kidney injury.^22^

The regulation of NTN‐1 in neuroinflammation can also be manifested by the mediation of PPARγ and NFκB. PPARγ, a cytoprotective protein, plays a pivotal in alleviating inflammatory responses.^23^ We showed that PPARγ level was decreased after SAH, and NTN‐1 treatment restored the PPARγ expression. In contrast, PPARγ inhibition with BADGE reversed the anti‐apoptotic effects of exogenous NTN‐1. Our study validated that PPARγ is a downstream effector of NTN‐1. Further, NFκB is a putative inflammation regulator by up‐regulating multiple pro‐inflammatory chemokines, cytokines, proteases, and adhesion molecules.^24^ The anti‐apoptotic effects of NTN‐1 concurred with down‐regulation of NFκB. Together, our findings indicate that the anti‐apoptosis effects of exogenous NTN‐1 originate at least in part through the regulation of PPARγ/NFκB signalling pathway after SAH. Notably, other factors, such as preservation of BBB integrity and enhanced neovascularization, may also contribute to the beneficial effects of NTN‐1. Further studies are in need to unravel other mechanisms of NTN‐1 treatment in SAH‐induced early brain injury.

## 5 CONCLUSIONS

In sum, here we demonstrated that NTN‐1 exerts potent neuroprotective effects in early brain injury induced by SAH, in which the UNC5B receptor and PPARγ are indispensable. Apoptosis was alleviated by NTN‐1 treatment and NFκB signalling was attenuated. NTN‐1 is a neuroprotective agent that can potentially improve the clinical management of early brain injury after SAH.

## CONFLICT OF INTEREST

None declared.
