# Supplementary figures and images for: Netrin‐1 alleviates subarachnoid haemorrhage‐induced brain injury via the PPARγ/NF‐KB signalling pathway
Source: J Cell Mol Med. 2019 Jan 7;23(3):2256–62. doi: 10.1111/jcmm.14105 (PMC6378208; doi:10.1111/jcmm.14105)

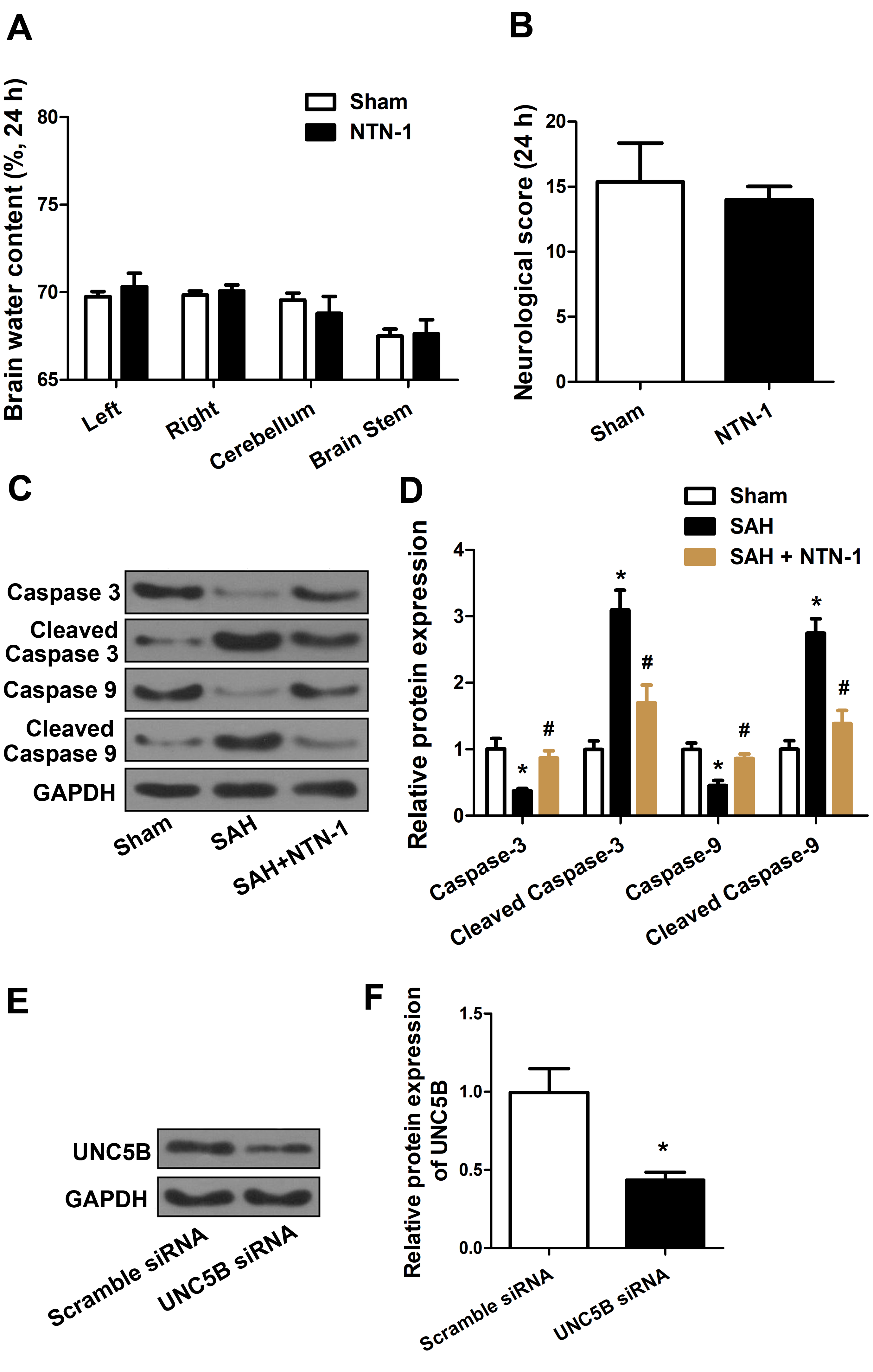

Supplement: Supplementary file 1 [file JCMM-23-2256-s001.tif]
